# Improvement of fibroid-associated symptoms and quality of life after US-guided high-intensity focused ultrasound (HIFU) of uterine fibroids

**DOI:** 10.1038/s41598-022-24994-w

**Published:** 2022-12-07

**Authors:** Tolga Tonguc, Florian Recker, Judith Ganslmeier, Holger M. Strunk, Claus C. Pieper, Olga Ramig, Simone Welz, Eva K. Egger, Nikola Mutschler, Leonie Warwas, Markus Essler, Alexander Mustea, Rupert Conrad, Milka Marinova

**Affiliations:** 1 Department of Diagnostic and Interventional Radiology, University Hospital Bonn, University Bonn, Venusberg-Campus 1, 53127 Bonn, Germany; 2 Department of Gynaecology and Gynaecological Oncology, University Hospital Bonn, University Bonn, Bonn, Germany; 3 Department of Nuclear Medicine, University Hospital Bonn, University Bonn, Bonn, Germany; 4 University Bonn, Bonn, Germany; 5 Department of Psychosomatic Medicine and Psychotherapy, University Hospital Muenster, Muenster, Germany

**Keywords:** Outcomes research, Urogenital reproductive disorders

## Abstract

Uterine fibroids are the most common benign uterine tumors and can cause various severe symptoms as abnormal menstrual bleeding or pelvic pain. Therefore, the primary objective in the treatment of uterine fibroids is a sufficient symptom relief. Ultrasound (US)-guided High-intensity focused ultrasound (HIFU) is an effective non-invasive treatment strategy for ablation of uterine fibroids that can achieve a significant tumor volume reduction. The aim of the study is to evaluate if US-guided HIFU treatment can reduce fibroid-associated symptoms leading to an improvement of health-related quality of life. Fifty-five women with symptomatic uterine fibroids underwent US-guided HIFU ablation. Clinical evaluation was performed on the basis of the Uterine Fibroid Symptom and Health-Related Quality of Life Questionnaire (UFS-QOL) at baseline, 6 weeks, 3, 6, 9 and 12 months after HIFU. Imaging follow-up included contrast-enhanced ultrasound (CEUS) and contrast-enhanced MRI. A significant reduction of the Symptom Severity Scale (SSS) was observed between 6 weeks and 12 months after HIFU (49.9 ± 19.4 at baseline vs. 42.2 ± 20.1 at 6 weeks and 23.6 ± 12.7 at 12 months after treatment, *p* < 0.001) correlating with a significant improvement (*p* < 0.001) of Health-related Quality of Life (HRQL) (52.5 ± 22.7 at baseline vs. 59.8 ± 22 at 6 weeks and 77.9 ± 17.3 at 12 months after treatment). Significant postinterventional improvement was observed in every subscale of HRQL. In the majority of patients, only minor, short-lasting and self-limiting side effects were observed, e.g. soft tissue edema of the anterior lower abdominal wall in the acoustic pathway or transient moderate lower abdominal pain as during menstruation. One patient with a very large fibroid experienced strong short-lasting pain after the procedure; two patients experienced post-procedurally a transient sciatic nerve irritation. US-guided HIFU of uterine fibroids reduces disease-related symptoms and improves health-related quality of life.

## Introduction

Uterine fibroids, also known as leiomyomas or myomas, are the most common benign uterine tumors and cause fibroid-associated symptoms in about 25–30% of the patients between 30 and 50 years^1–4^. A major symptom is abnormal menstrual bleeding, in particular severe and extended bleeding (hypermenorrhoea and menorrhagia) that can be associated with pelvic pain during menstruation (dysmenorrhea) and cause subsequent anemia^4–6^. Large tumors can cause pelvic pressure or pain and compress adjacent organs which can lead to urinary urgency and constipation^6,7^. Further, uterine fibroids can be associated with infertility and pregnancy complications^5,6^.

Current treatment strategies mainly involve surgical interventions as laparoscopic or hysteroscopic myomectomy and laparoscopic hysterectomy^5,8,9^. At the same time minimally invasive and non-invasive therapies are becoming increasingly important^5,10,11^. In animal data it has been shown that High-Intensity Focused Ultrasound (HIFU) is an effective method for non-invasive ablation of uterine fibroids as it results in significant reduction of fibroid volumes^12^. Tumor volume reduction over time after HIFU treatment is due to a coagulation necrosis in the ablated area which is based on thermal ablation^13–15^. This is enhanced by physical effects as acoustic cavitation^16^. Both Magnetic Resonance (MR)-guided and Ultrasound (US)-guided HIFU are suitable for therapy of symptomatic patients^10,17–20^. Long-term clinical data show that HIFU can induce fibroid ablation with subsequent lesion shrinkage in patients with symptomatic uterine fibroids^10,11,21–23^. Moreover, there is clinical evidence of improvement in disease-related symptoms after HIFU in combination with shorter hospital stays, fewer adverse events and faster recovery than laparoscopic myomectomy, as well as further data suggesting that HIFU may improve the patients’ health-related quality of life^10,17,20,24^.

As more data on patient-relevant clinical outcome following HIFU treatment are needed, the aim of this study was to investigate changes in fibroid-associated symptoms and their impact on the quality of life of treated patients. The primary objective therefore relates to patient-reported outcome using the Uterine Fibroid Symptoms and Quality of Life (UFS-QOL) questionnaire.

## Methods

### Patient selection and characteristics

Data of 55 patients with uterine fibroids (average age 43.4 years, Table 1) treated with US-guided HIFU were analyzed. Diagnostic ultrasound and MRI of the pelvis were used to decide on fibroid eligibility for US-guided HIFU. Indications for HIFU treatment were confirmed by an interdisciplinary board in consideration of inclusion and exclusion criteria (Table 2). The study was performed in accordance with the Declaration of Helsinki and approved by the local ethics committee of the University Hospital Bonn. Written informed consent was obtained from all patients, inclusive for using their data. Clinical indications for US-guided HIFU treatment were based on leading symptoms as hypermenorrhea and dysmenorrhea, pelvic pain and frequent urination (Table 1).

**Table 1:** Clinical characteristics of HIFU-treated patients with uterine fibroids.

Parameter	
Number of patients (mean)	55
Age (years; mean)	43.4 ± 6.3 (range 28–57)
BMI (mean)	23.2 ± 3.6 (range 16.4–36.4)
**Leading symptoms**	
Hypermenorrhea and dysmenorrhoea	50 (91%)
Pelvic pain	39 (71%)
Frequent urination	38 (69%)
Fatigue	51 (92%)
**Localisation of uterine fibroids**	
Anterior wall	22 (40%)
Posterior wall	15 (27%)
Anterior and posterior wall	12 (22%)
Lateral wall	6 (11%)
Mean initial volume of treated fibroids	109.9 ± 125 ml (range 4.5–634.2)
Desire for children	30 (54%)
**Previous gynaecological treatment**	
Oral contraception	7
Ulipristal acetate	9
Myomectomy	5
Non-therapeutic hysteroscopy	2
Cesarean section	3

**Table 2:** Inclusion and exclusion criteria for US-guided HIFU.

Inclusion criteria	Exclusion criteria
Age ≥ 18 years	Extensive scarring in the acoustic pathway
Written informed consent	Calcified fibroids
Fibroid-related symptoms	Mesh materials in the anterior inferior abdominal wall after hernia repair
Suitability for sedation	
Visualisation in diagnostic sonography	
Accessibility (distance between skin and deepest fibroid area max. 11 cm)	
Fibroid diameter ≥ 2 cm	

### US-guided HIFU treatment

**Figure 1:**
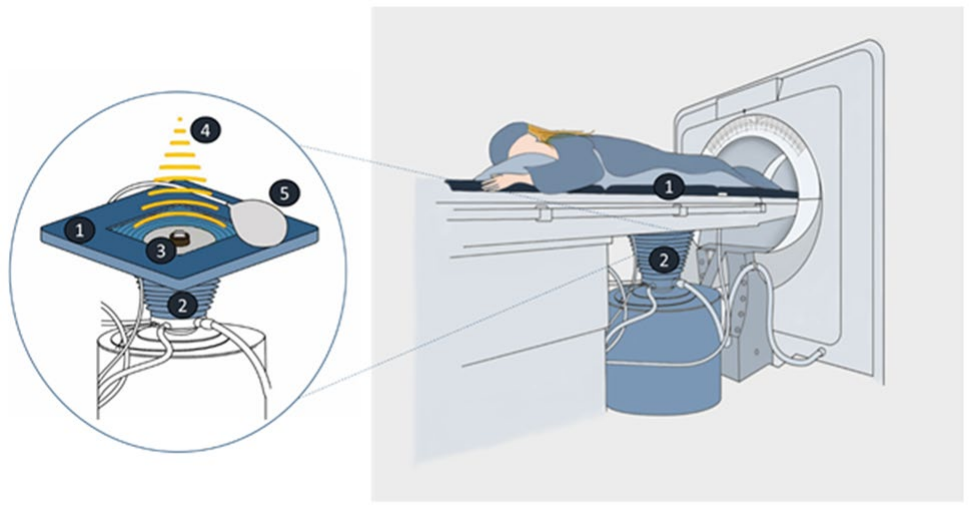
US-guided HIFU system: schematic illustration of the US-guided HIFU unit JC Tumor Therapeutic System (HAIFU Medical Technology, Chongqing, China). The treatment table has an aperture (1) with a water basin (2). Patients with uterine fibroids lie in prone position with their lower abdominal wall at the level of the aperture, so that the skin gets into contact with cooled water. The parabolic therapeutic transducer (3) and the convex diagnostic ultrasonography probe are located in the center of the water basin. A therapeutic ultrasound beam (4) is transmitted to the lesion. A ceramic transducer with a diameter of 15 cm and a focal length of 15 cm is used for this at a frequency of 0.8 MHz. Between the transducer and skin of the lower anterior abdominal wall a balloon filled with degassed water (5) is positioned to keep the bowel away from the acoustic pathway. The ultrasound beam generated during the HIFU procedure has an oval-shaped focus of 1-3 mm in width and 8-15 mm in length. In this approach, a coagulation necrosis lesion is created in the target area. The next focal point is selected when grey-scale changes become apparent in the target area indicating effective ablation. Thus, multiple adjacent lesions on bordering layers produce linear and discoidal necrosis patches, allowing the entire tumor area to be ablated as a volume unit.

The day before the intervention, patients were asked to complete a bowel preparation regimen that included a 12-h fast and the use of laxatives which aims protection from heat-induced adverse effects. Immediately before HIFU procedure, the skin in the acoustic pathway was prepared by shaving, degreasing, and degassing the anterior lower abdominal wall. HIFU ablation was performed under conscious sedation using the Focused Ultrasound Tumor Therapeutic System (JC TTS, Haifu Medical Technology, Chongqing, China) (Fig. 1).

Target fibroids were identified with diagnostic ultrasound, sagittal slices were used to create a sonication plan. A safety distance of 1 cm to the fibroid margin and surrounding structures at risk (bowel, biliary stents) was maintained to prevent heat-induced complications. The volume ablation was achieved by performing multiple focal sonications in rows and adjacent layers. The applied power was adjusted individually for every patient (Table 3).

**Table 3:** Intervention parameters of the HIFU treatment.

Parameter	Value
Treatment time [min]	162 ± 47^#^
Sonication time [s]	1089 ± 429
Total energy [kJ]	307.62 ± 146.7
Average power [W]	278 ± 68

^#^Mean ± standard deviation values are shown.

Adverse events were recorded by the interventionist and nurses at each follow-up time point (1, 6 weeks, 3, 6, 9 months, 1 year after HIFU). If a treatment-related adverse event was observed, patients were called at two-week intervals until the event had resolved.

### Clinical evaluation (UFS-QOL-Questionnaire)

Clinical evaluation after HIFU treatment was performed using the Uterine Fibroid Symptom and Health Related Quality of Life Questionnaire (UFS-QOL). The UFS-QOL consists of 37 questions in total out of which 8 items refer to the Symptom Severity Scale (SSS) and 29 items are specific for the patients’ Health-Related Quality of Life (HRQL). The first 8 items refer to fibroid-associated symptoms as changes during menstruation, pelvic pain, frequent urination and fatigue. Subscales of HRQL are Concern, Activities, Energy and Mood, Control, Self-consciousness and Sexual Function. All items were scored on a Likert-scale from 1 to 5 points. The sums of the subscale scores were converted into a transformed score for symptom severity and HRQL ranging from 1 to 100. Consequently, a high score on the Symptom Severity Scale indicates greater symptoms, whereas a high score for HRQL and its subscales indicates a better quality of life^24–26^. Assessment with UFS-QOL was performed at baseline prior to the intervention and within the first post-ablation week, after 6 weeks and 3 months followed by 3-month time intervals.

### Follow-up imaging

The follow-up imaging consisted of contrast-enhanced MRI (1.5- T. Ingenia MRI, Philips Healthcare, Best, the Netherlands) and CEUS (Ultrasound device: EPIQ 5G, Philips Healthcare, Best, the Netherlands; Contrast medium: SonoVue, Bracco Imaging S.p.A., Milan, Italy). CEUS was used for verification of an effective US-guided ablation and MRI to evaluate treatment success in detail by visualizing the non-perfused ablation volume of the target fibroids. The first post-interventional imaging was performed as follows: CEUS examination immediately after the HIFU procedure, MRI within the next 4 days.

### Statistical analysis

Data were analyzed using Stata Version 14.2 (StataCorp. Stata Statistical Software: Release 14. College Station, TX: StataCorp LP). Mean, median, standard deviation (SD), range, and 95% confidence intervals (CI) were estimated. The Symptom Severity Score and multivariate dimensions of health-related QOL (energy and mood; control; concern; activities; sexual function; self-consciousness) were assessed using the UFS-QOL questionnaire. The statistical evaluation of SSS and QOL subscales was performed using the mixed longitudinal (panel) model at baseline and each follow-up as dependent variable. Follow-up questionnaires were available at the time points 1 and 6 weeks, 3, 6, and 9 months, 1 year post-HIFU. Results were presented as contrasts (differences) and were verified for susceptibility of model dependency using a non-parametric Skilling-Mack test for unbalanced panel data. A value of < 0.05 was considered statistically significant.

## Results

### HIFU treatment

**Figure 2:**
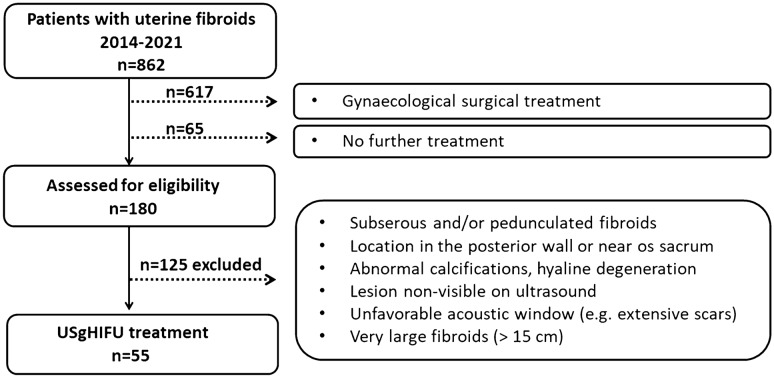
Study consort diagram: assessment for eligibility.

**Figure 3:**
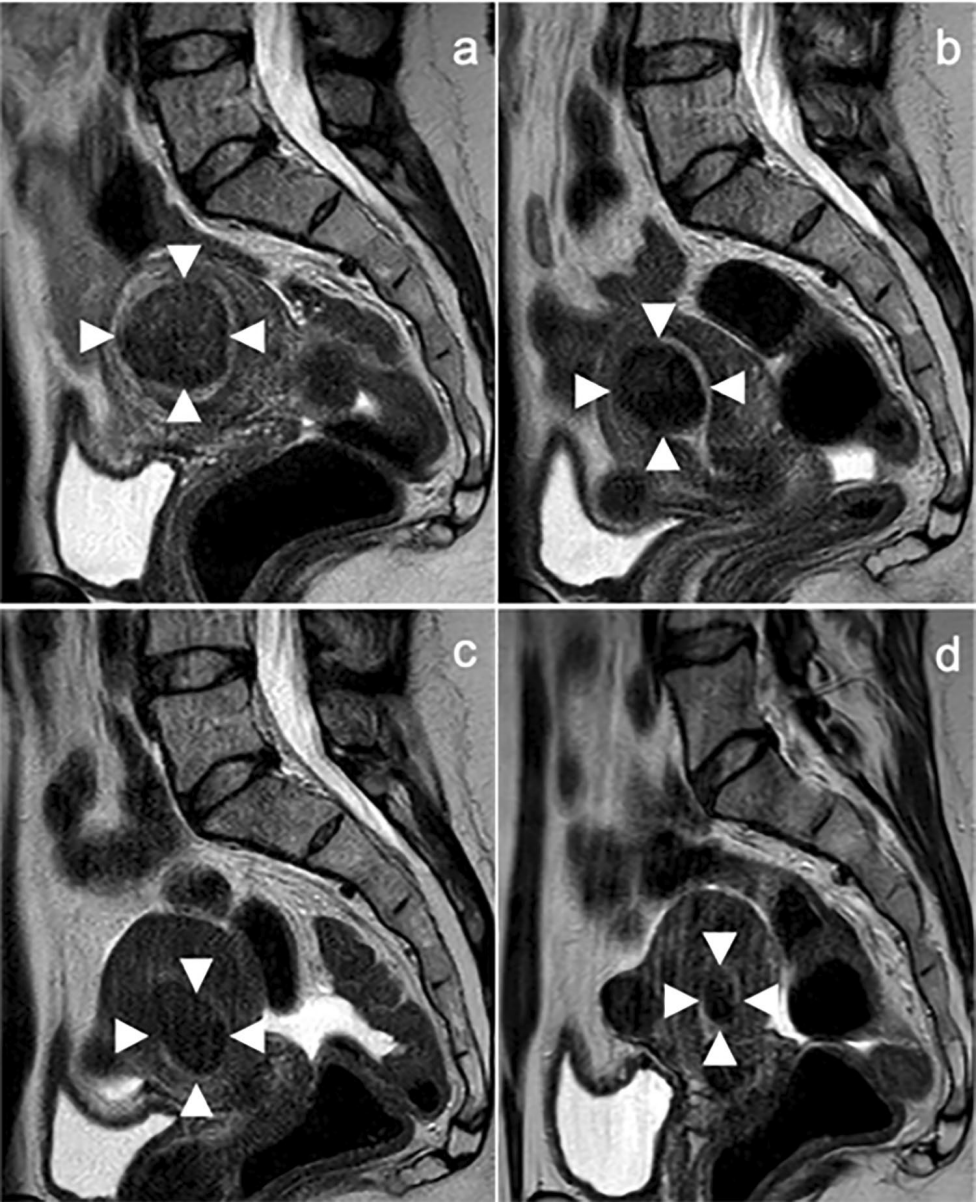
MRI-images (sagittal T2-weighted sequences) of a 41-yearS old premenopausal patient with multiple fibroids who presented with severe fibroid-associated symptoms (hypermenorrhea, dysmenorrhea, anaemia, pelvic pain and pressure). There was no fibroid-specific previous therapy. The largest fibroid was located in the fundus of the uterus and was the main target of ablation. In the 9-month follow-up a significant reduction of tumor volume was observed. (**a**) Large predominant target myoma in the uterine fundus before HIFU treatment. (**b**) At 3-month follow-up the volume reduction was about 53.7%. (**c**), (**d**) Fibroid volume reduction of about 87.8% after 6 months (**c**) and 92.7% after 9 months (**d**) post-HIFU.

About 72% of patients with symptomatic uterine fibroids at our center underwent gynaecological surgical treatment (617/862). Eligibility for US-guided HIFU was evaluated in 180 patients, 31% (55/180) were suitable for treatment (Fig. 2). The treatment was performed successfully in selected patients (n = 55, 54 Caucasian, 1 African) regardless of fibroid location and initial lesion volume (e.g. Figure 3). Intervention parameters are summarized in Table 3. Most patients (50/55) were treated with a single treatment session, only 5 patients (9%) underwent a second HIFU ablation due to persistence of symptoms with a minimum interval of 7 months to the first treatment.

In the majority of patients, only minor, short-lasting and self-limiting side effects were observed, e.g. soft tissue edema of the anterior lower abdominal wall in the acoustic pathway (17/55) or transient moderate lower abdominal pain as during menstruation (12/55). One patient with a very large fibroid experienced strong short-lasting pain after the procedure; two patients experienced post-procedurally a transient sciatic nerve irritation.

### Clinical evaluation (symptom severity score and health-related quality of life)

**Figure 4:**
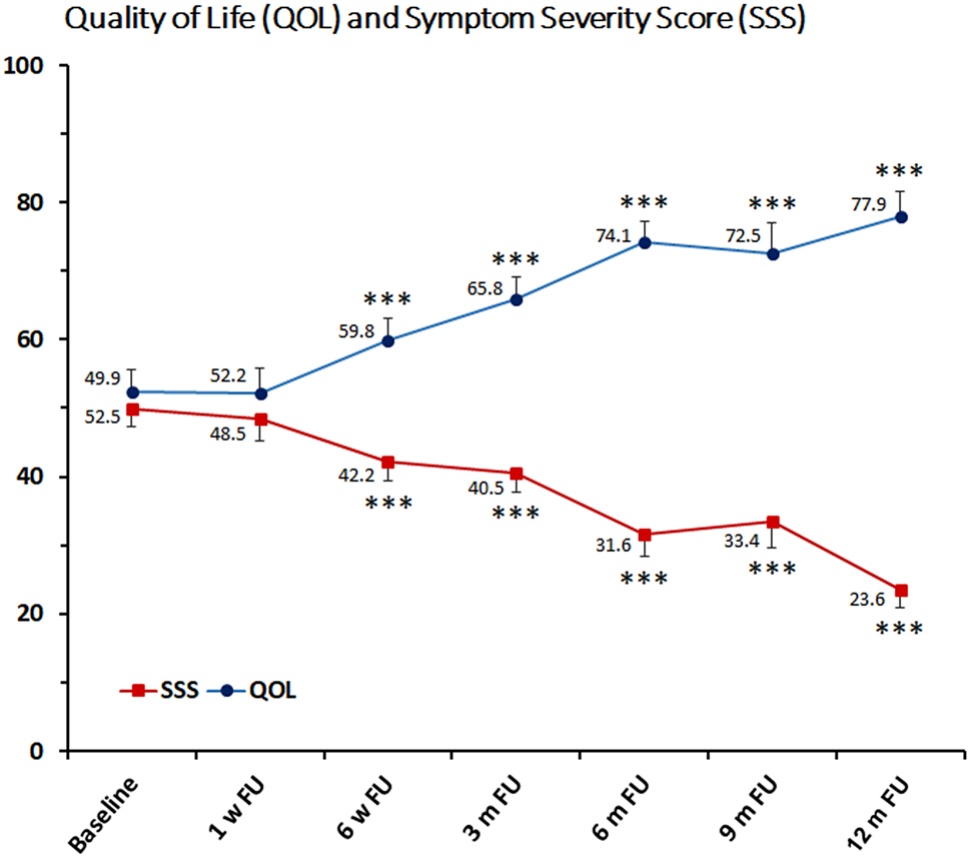
Mean scores of Symptom Severity Scale (SSS) and Health-Related Quality of Life (HRQOL) after ablation of uterine fibroids with US-guided HIFU. Significance levels are indicated by the stars attached (*** indicating *p* < 0.001) next to average scores. Standard errors were symmetrical (positive error bars shown for HRQOL and negative bars shown for SSS). *FU* Follow-up, *m* month, *w* week.

**Figure 5:**
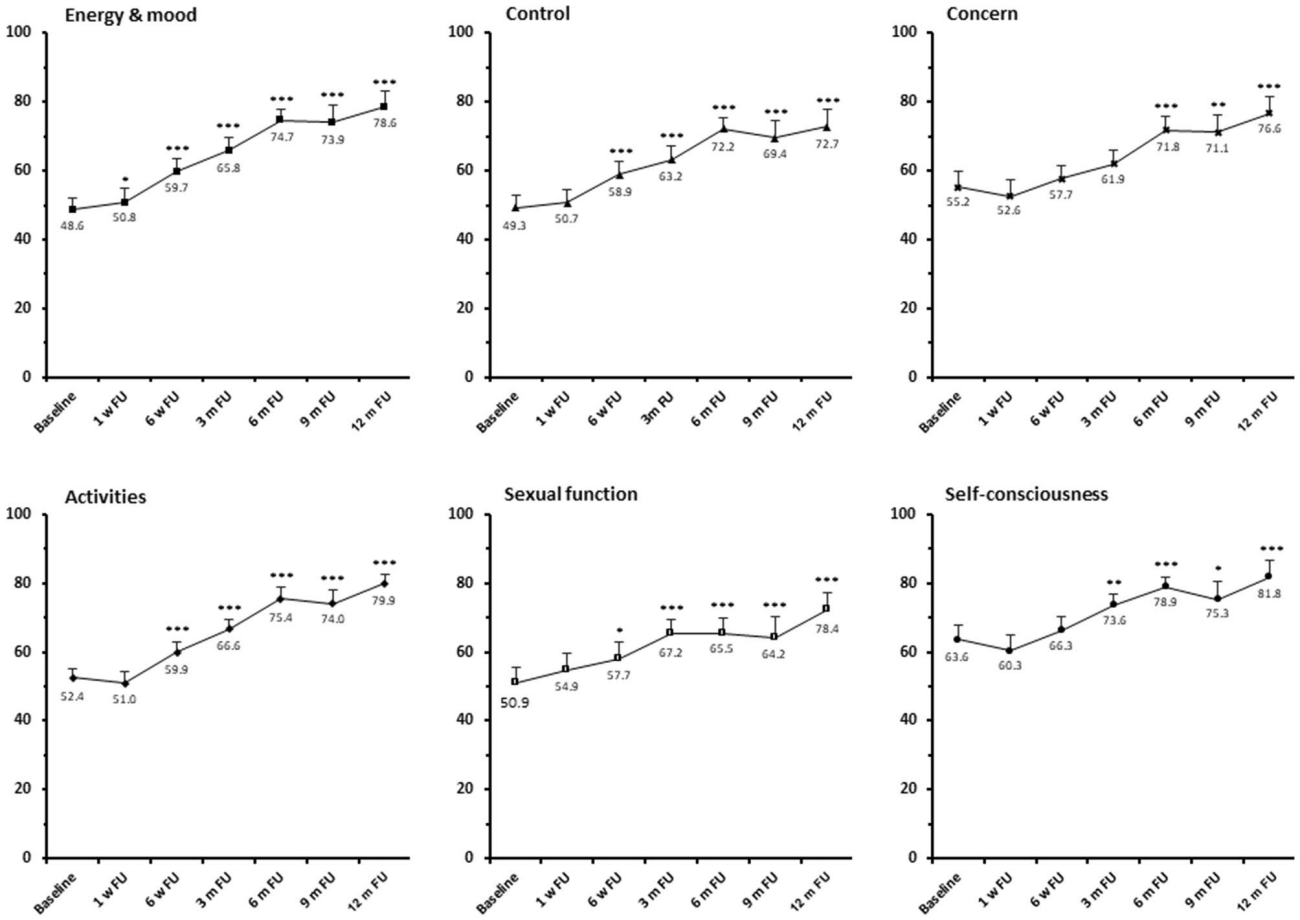
Mean scores of the subscales of Health-Related Quality of Life (HRQOL) after HIFU ablation of uterine fibroids are presented. Significance levels are indicated by the stars attached (*p < 0.05, **p < 0.01, ***p < 0.001) next to average scores. Standard errors were symmetrical (only positive error bars are shown). The average postinterventional scores indicate a significant treatment-associated improvement of all QOL subcales (energy&mood, control, concern, activites, sexual function, self-consciousness). *FU* Follow-up, *m* month, *w* week.

The clinical follow-up showed a significant reduction of fibroid-associated symptoms and a consequent improvement of quality of life. A significant decline of the mean Symptom Severity Score (SSS) was observed between 6 weeks and 12 months after ablation (49.9 ± 19.4 at baseline vs. 42.2 ± 20.1 at 6 weeks vs. 23.6 ± 12.7 at 12 months after treatment) correlating with a significant increase of the Health-Related Quality of Life (HRQOL) between 6 weeks and 12 months after HIFU (52.5 ± 22.7 at baseline vs. 59.8 ± 22 at 6 weeks vs. 77.9 ± 17.3 at 12 months after treatment) (Fig. 4). Considering the different items of HRQOL a significant improvement of the subscale Energy/Mood was observed between one week and 12 months after HIFU (48.6 ± 26.6 at baseline vs. 78.6 ± 21.2 at 12 months after treatment). An improvement of physical activities was represented by a significant increase of the mean scores of the subscales Activities and Sexual Function between 6 weeks and 12 months after ablation (Activities: 52.4 ± 27.1 at baseline vs. 79.9 ± 20.4 at 12 months after treatment; Sexual Function: 50.2 ± 32.9 at baseline vs. 78.4 ± 23.2 at 12 months after treatment). Another ablation-associated effect was the improvement of the patients’ psychological well-being indicated by a significant score increase of the subscales Control between 6 weeks and 12 months (49.4 ± 24.7 at baseline vs. 72.7 ± 23.3 at 12 months after treatment), Self- consciousness between 3 and 12 months (63.5 ± 29.5 at baseline vs. 81.8 ± 22.5 at 12 months) and Concern between 6 and 12 months (55.2 ± 32.7 at baseline vs. 72.7 ± 5 at 12 months after treatment) (Fig. 5).

## Discussion

The development of self-reported quality of life showed a nearly steady rise of the HRQOL score corresponding to a steady decline of the SSS score. The validity of our findings is affirmed by the fact that the Uterine Fibroid Symptom and Quality of Life (UFS-QOL) questionnaire is a psychometrically sound outcome measure, which is recommended to assess patient outcome over time and across treatments^24,27–29^. It does not only assess overall quality of life and symptom severity, but also gives a differentiated insight into specific aspects of physical and psychological well-being.

The subdomain Activities ask for physical, social or other daily activities, whereas the subdomain Energy/Mood assesses tiredness, anxieties and sadness or hopelessness. Our findings show convincingly the significant stabilization of mood and vitality and increased ability to take part in all kinds of daily activities. Mean scores for both subdomains reached over 70 points, which indicates that at the 12-months follow-up symptom-related limitations with regard to activities and mood existed on average only between a little and some of the time^27^. As the most important target of treatment is sufficient control of symptom burden the subdomain Control is of specific relevance. As early as 6 weeks after the intervention women reported significant improvements on control persisting until the 12-months follow-up. This is of particular clinical relevance as symptomatic uterine fibroids often grossly interfere with a wide spectrum of activities negatively affecting patients’ conviction of being in control.

The dimension Concern asks for general health-related concerns and more specifically for concerns regarding soiling of clothes or linen showing a delayed but significant improvement between 6 and 12 months after HIFU. Moreover, HIFU has a positive impact on the subdomain Self-consciousness that assesses critical self-awareness and attention regarding physical appearance and improves the patients’ sexual function showing that HIFU treatment improves fibroid-associated physical restrictions and reduces the disease-related psychological burden.

In line with our results a current meta-analysis including 4450 women compared HIFU with surgical interventions for symptomatic uterine fibroids proving the superiority of HIFU regarding symptomatic relief and improvement of quality of life at 6- and 12-month follow-up, duration of hospital stays and time to return to work^30–34^. There were no significant differences in terms of symptom recurrence, re-interventions and pregnancy^30^.

Clinical data based on UFS-QOL show symptomatic improvement after HIFU treatment of uterine fibroids which corresponds to an equally convincing increase in quality of life^32,35–40^. However, regarding outcomes, apart from two studies^35,36^, above mentioned studies only report the SSS score and the total HRQOL score, making a more differentiated assessment of the development of quality of life difficult. The mentioned studies by Kim et al.^35^ and Ikink et al.^36^ excluded women, who wanted to become pregnant after the treatment. Furthermore, the study of Kim et al. did not report ethnicity of the patient sample, therefore we confined our comparison to the study by Ikink et al.^36^, who investigated 41 patients (39 Caucasian) over a period of 6 months. In our study, 55 patients (54 Caucasian, 1 African) were followed up over a one-year period.

Total HRQOL score showed a significant increase equalling 15.2 points compared to 24.2 in our study after 6 months. Taking a closer look at the six subscales, the subscale Concern showed a 14.6 point increase (16.6 in our study), Activities 14.1 points (23), Energy/Mood 15.0 (26.1), Control 10.3 (22.9), Self-consciousness 18.9 (15.3), and Sexual function 10.4 (14.6) points. The comparison shows that particularly with regard to the subscales Activities, Energy/Mood, and Control our study group showed a clinically relevant higher increase. As the study by Ikink et al. dates back about 8 years it is difficult to explain these differences in efficacy at the 6-months follow-up. On the one hand, the patient sample of Ikink et al. had higher baseline QOL. On the other hand, among other factors, advances in HIFU technology that allow for more effective ablation compared to the older ablation protocol of Ikink et al. or the HIFU approach used (US-guided vs. MR-guided) might be associated with these findings.

The study was limited by a moderate sample size not allowing the detection of small effects on quality of life and limited external validity of findings due to data from a single tertiary care center. Another methodological limitation is related to the uterus-preserving nature of HIFU treatment, so that fibroids and symptoms may return. In addition, our patients were followed up for up to one year, which represents mid-term outcome. In the long term, it could be possible that symptoms return and quality of life decreases again.

In summary, our findings confirm convincingly the efficacy of HIFU on all aspects of quality of life in women suffering from uterine fibroids. The treatment supports the patients’ self-efficacy, stabilizes their mood and energy and counteracts functional impairment. A differentiated assessment of quality of life after HIFU treatment and minimally-invasive interventions is the precondition for an optimal individual treatment of uterine fibroids.

## Data Availability

Datasets generated and analyzed as part of this study are available upon request from the corresponding author at any time.
